# Identification of the immune-related biomarkers in Behcet’s disease by plasma proteomic analysis

**DOI:** 10.1186/s13075-023-03074-y

**Published:** 2023-06-01

**Authors:** Huan Liu, Panpan Zhang, Fuzhen Li, Xiao Xiao, Yinan Zhang, Na Li, Liping Du, Peizeng Yang

**Affiliations:** 1 Department of Ophthalmology, The First Affiliated Hospital of Zhengzhou University, Henan Province Eye Hospital, Henan International Joint Research Laboratory for Ocular Immunology and Retinal Injury Repair, Jianshe East Road 1, Zhengzhou, 450052 Henan Province People’s Republic of China; 2 The Academy of Medical Sciences, Zhengzhou University, Zhengzhou, 450052 Henan Province People’s Republic of China; 3 Department of Rheumatology and Immunology, The First Affiliated Hospital of Zhengzhou University, Zhengzhou, 450052 Henan Province People’s Republic of China; 4 The First Affiliated Hospital of Chongqing Medical University, Chongqing Key Laboratory of Ophthalmology and Chongqing Eye Institute, Youyi Road 1, Chongqing, 400016 People’s Republic of China

**Keywords:** Behcet’s disease, Uveitis, Immune, Proteomics, Biomarker

## Abstract

**Background:**

This study aimed to investigate the expression profile of immune response-related proteins of Behcet’s disease (BD) patients and identify potential biomarkers for this disease.

**Methods:**

Plasma was collected from BD patients and healthy controls (HC). Immune response-related proteins were measured using the Olink Immune Response Panel. Differentially expressed proteins (DEPs) were used to construct prediction models via five machine learning algorithms: naive Bayes, support vector machine, extreme gradient boosting, random forest, and neural network. The prediction performance of the five models was assessed using the area under the curve (AUC) value, recall (sensitivity), specificity, precision, accuracy, F1 score, and residual distribution. Subtype analysis of BD was performed using the consensus clustering method.

**Results:**

Proteomics results showed 43 DEPs between BD patients and HC (*P* < 0.05). These DEPs were mainly involved in the Toll-like receptor 9 and NF-κB signaling pathways. Five models were constructed using DEPs [interleukin 10 (IL10), Fc receptor like 3 (FCRL3), Mannan-binding lectin serine peptidase 1 (MASP1), NF2, moesin-ezrin-radixin like (MERLIN) tumor suppressor (NF2), FAM3 metabolism regulating signaling molecule B (FAM3B), and O-6-methylguanine-DNA methyltransferase (MGMT)]. Among these models, the neural network model showed the best performance (AUC = 0.856, recall: 0.692, specificity: 0.857, precision: 0.900, accuracy: 0.750, F1 score: 0.783). BD patients were divided into two subtypes according to the consensus clustering method: one with high disease activity in association with higher expression of tripartite motif-containing 5 (TRIM5), SH2 domain-containing 1A (SH2D1A), phosphoinositide-3-kinase adaptor protein 1 (PIK3AP1), hematopoietic cell-specific Lyn substrate 1 (HCLS1), and DNA fragmentation factor subunit alpha (DFFA) and the other with low disease activity in association with higher expression of C–C motif chemokine ligand 11 (CCL11).

**Conclusions:**

Our study not only revealed a distinctive immune response-related protein profile for BD but also showed that IL10, FCRL3, MASP1, NF2, FAM3B, and MGMT could serve as potential immune biomarkers for this disease. Additionally, a novel molecular disease classification model was constructed to identify subsets of BD.

**Supplementary Information:**

The online version contains supplementary material available at 10.1186/s13075-023-03074-y.

## Introduction

Behcet’s disease (BD) is a chronic, multisystem autoinflammatory disorder characterized by recurrent oral and genital ulcerations, uveitis, and skin lesions, as well as vascular, neurological, and gastrointestinal manifestations [1, 2]. BD, also called Silk Road disease or Behcet’s syndrome, mainly occurs in countries along the ancient Silk Road from the Mediterranean Basin across Asia to Japan [3]. BD is considered as one of the most common causes of uveitis and the primary cause of blindness [4]. Our recent study involving 15 373 uveitis patients showed that BD accounted for 10.6% of cases [5].

Although the etiology of BD remains unclear, genetic susceptibility, environmental factors, viral and bacterial infections, inflammation, and immune dysregulation are involved in its development [6–9]. Immune dysfunction of both adaptive and innate immunity plays an essential role in the pathogenesis and progression of BD [10]. The levels of pro-inflammatory and anti-inflammatory cytokines have been extensively studied in the serum and plasma of patients with BD [11–13]. These inflammatory cytokines produced by immune cells can regulate or activate other immune cells, causing tissue damage. For example, T helper 17 (Th17) cells, which are the major subsets of CD4^+^ T cells, are essential to the process of BD. The differentiation of human naïve CD4^+^ T cells into Th17 cells is regulated by cytokines such as interleukin 6 (IL 6), transforming growth factor-β (TGF-β), interleukin 21 (IL 21), and interleukin 23 (IL 23) [14, 15]. Several studies have demonstrated that cytokines could serve as potential drug targets for the treatment of BD or candidate biomarkers for the prediction of disease activity, severity, and prognosis [16–18].

Evidences also indicate that some immune response-related proteins can regulate the secretion of inflammatory cytokines and differentiation of immune cells via the Janus kinase—signal transducer and activator of transcription (JAK-STAT), nuclear factor-κB (NF-κB), and P38 mitogen-activated protein kinase (P38-MAPK) signaling pathway [19–21]. For example, increased tripartite motif-containing 21(TRIM21) can activate the NF-κB signaling pathway to promote the secretion of IL6, interleukin 1β (IL 1β), and IL 23 and induce the differentiation of Th17 cells in BD [22]. However, the potential role of immune response-related proteins in immune and inflammatory function modulation in BD is less well studied.

The aim of this study was to investigate the expression profile of immune response-related proteins in the plasma of patients with BD and identify potential plasma biomarkers in BD.

## Methods

### Study population

Active BD patients ((training cohort *n* = 27, validation cohort *n* = 28) and healthy controls [HC (training cohort *n* = 25, validation cohort *n* = 28)] matched by age and sex were enrolled in the study from the First Affiliated Hospital of Zhengzhou University. BD was strictly diagnosed by rheumatologists according to the diagnostic criteria developed by the International Study Group for Behçet’s disease [1]. BD activity was evaluated using the Behçet Disease Current Activity Form (BDCAF) [23]. Uveitis was diagnosed by an ophthalmologist. Intraocular inflammation was evaluated according to the standardized uveitis nomenclature (SUN) working group classification [24]. Detailed demographic information and clinical details of the BD patients are listed in Table 1 and Supplementary Table S1.

**Table 1:** Clinical characteristics of Behcet’s disease (BD) and healthy controls (HC)

	**Overall**	**Female**	**Male**	***p. value***
HC				
HC (*N*, %)	25	12 (48.00)	13 (52.00)	
Age (mean (SD))	40.28 (8.11)	41.67 (8.54)	39.00 (7.81)	0.423
BD				
BD (*N*, %)	27	13 (48.10)	14 (51.90)	
Age (mean (SD))	33.37 (14.19)	36.92 (17.70)	30.07 (9.44)	0.216
Treatment (%)	8 (29.60)	2 (15.40)	6 (42.90)	0.322
Disease duration (months) (median (Q1, Q3))	36.00 (21.50–74.00)	36.00 (24.00–40.00)	68.50 (20.25–75.00)	0.593
Oral or Genital ulcers (%)	27 (100.00)	13 (100.00)	14 (100.00)	
Skin involvement (%)	8 (29.60)	4 (30.80)	4 (28.60)	1.000
Joint involvement (%)	7 (25.90)	4 (30.80)	3 (21.40)	0.909
Uveitis (%)	14 (51.90)	7 (53.80)	7 (50.00)	1.000
Vascular involvement (%)	4 (14.80)	0 (0.00)	4 (28.60)	0.122
Neurological involvement (%)	2 (7.40)	2 (15.40)	0 (0.00)	0.430
Gastrointestinal involvement (%)	2 (7.40)	1 (7.70)	1 (7.10)	1.000

Treatment: GCs or other immunosuppressive agents

### Plasma collection

Fresh peripheral blood (10 ml) was collected in EDTA tubes, and plasma was isolated by centrifugation at 2000 g for 10 min, and then stored at − 80 °C until use.

### Measurement of plasma proteins

The plasma levels of 92 immune response-related proteins were measured using a proximity extension assay (PEA, Olink Proteomics, Shanghai, China) [25]. The data are presented as normalized protein expression (NPX) values on a log2 scale. Twelve proteins were excluded from downstream analysis with intra- and inter-assay coefficient of variance (%CV) and the frequency of missing values of more than 20% in each sample. One patient sample was excluded because of quality control failure (Supplementary Figure S1a). In addition, an NPX value of less than 0 was replaced by the intragroup mean in some samples.

### Data analysis and statistics methods

Principal component analysis (PCA) was performed using “FactoMiner” and “factoextra” R packages. Categorical variables are described as numbers (percentages) and compared using the chi-square test or Fisher’s exact test. Continuous variables are presented as median and interquartile range (IQR). Differences between two and three groups were compared using the non-parametric Mann–Whitney *U* test and Kruskal–Wallis test with Dunn’s correction, respectively. The results are presented in the form of tables or boxplots. Volcano and heatmap plots were drawn using the “ggpubr” and “pheatmap” packages. The correlated heatmap was plotted to visualize the Pearson’s correlations between differentially expressed proteins (DEPs) using the “ggcorrplot” package.

### Bioinformatics analysis

Gene ontology (GO) and the Kyoto Encyclopedia of Genes and Genomes (KEGG) enrichment analyses were performed using the R package “clusterProfiler” (version 3.18.1) [26]. To further investigate the correlation between DEPs, a protein network interaction diagram (PPI) was constructed using the online tool STRING (version 11.5, https://cn.string-db.org/).

### Feature selection and prediction model creation

The recursive feature elimination (RFE) algorithm, which includes feature extraction, feature selection, and model training, was performed for the features selected based on the random forest (RF) with fivefold repeated cross-validation. All DEPs were used to train the prediction model, and the feature importance of the variables was calculated and ranked using accuracy and kappa metrics. An optimal subset of features was selected from all DEPs for the prediction model creation. To construct the prediction model, five algorithms were used based on the package “caret”: naive Bayes (NB), support vector machine (SVM), extreme gradient boosting (XGB), random forest (RF), and neural network (NNET). Receiver operating characteristic (ROC) analysis, recall (sensitivity), specificity, precision, accuracy, F1 score, and residual distribution were used to assess the prediction performance of the different models on the testing set. ROC curves were plotted using the “pROC” package.

### Consensus clustering

To investigate the role of differentially expressed immune response-related proteins, different clinical phenotypes, sex, and age in BD patients, K-means consensus clustering with k from 2 to 7 was performed using the R package “ConsensusClusterPlus.” The clustering results were visualized using t-distributed stochastic neighbor embedding (tSNE) based on the “Rtsne” R package. All analyses were carried out using the R language, version 4.0.3.

## Results

### DEPs between BD and HC

**Fig. 1:**
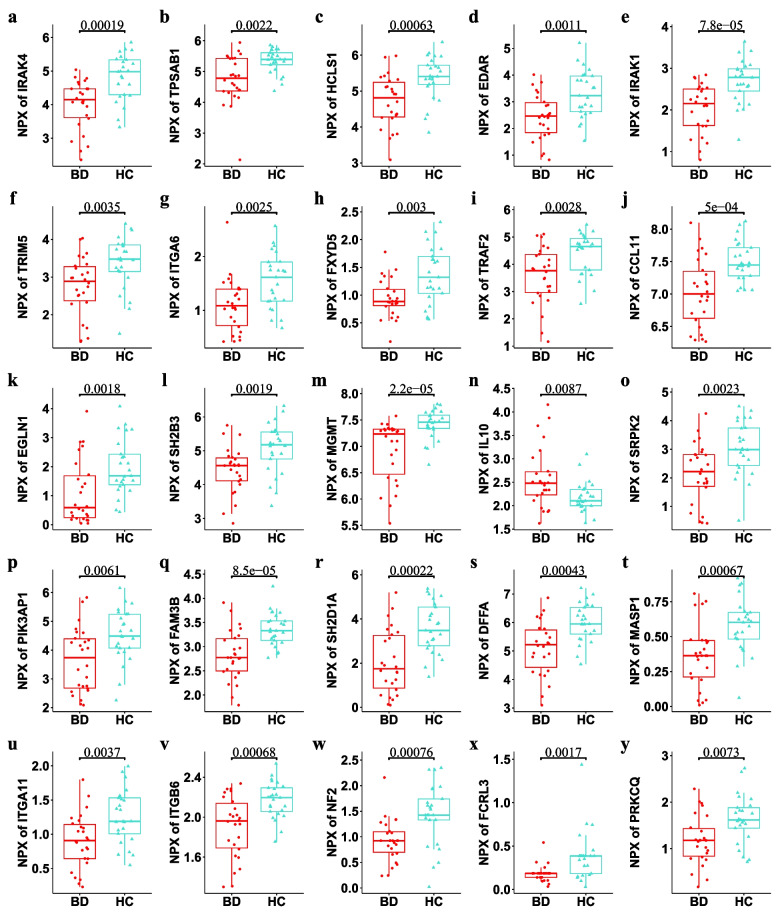
Boxplots were made to present the top 25 differential immune-related proteins between Behcet’s disease and healthy controls

**Fig. 2:**
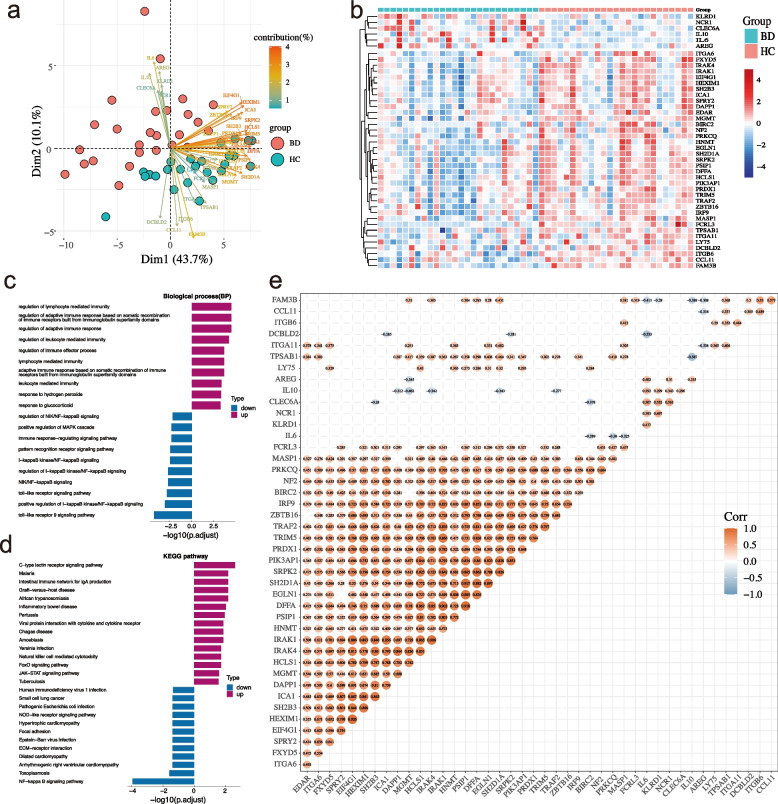
Bioinformatics analysis of differentially expressed proteins of Behcet’s disease. **A** PCA is based on 43 differentially expressed proteins (DEPs). **B** Clustering heatmap of DEPs. **C** GO (BP) enrichment analysis. **D** KEGG enrichment analysis of differential proteins. **E** Correlation heatmap between DEPs

To identify DEPs in the plasma of BD patients and HC, 92 immune response-related proteins were measured using a PEA. The expression of 43 immune response-related proteins differed between BD patients and HC (*P* < 0.05). Six proteins were upregulated and 37 proteins were downregulated in our study. The upregulated proteins included IL6, IL10, killer cell lectin like receptor D1 (KLRD1), natural cytotoxicity triggering receptor 1 (NCR1), amphiregulin (AREG), and C-type lectin domain-containing 6A (CLEC6A) and the downregulated proteins mainly included MGMT, interleukin 1 receptor-associated kinase 1 (IRAK1), FAM3B, interleukin 1 receptor-associated kinase 4 (IRAK4), SH2D1A, and DFFA. All the DEPs are listed in Supplementary Table S2. Boxplots were constructed to represent the top 25 DEPs (Fig. 1). PCA based on the 43 DEPs was able to discriminate BD from HC samples (Fig. 2a). DEPs were visualized using a volcano plot (Supplementary Figure S1b). A clustering heatmap was plotted to show the DEPs in the different samples (Fig. 2b).

### GO and KEGG pathway analysis

Bioinformatics analysis was performed to investigate the biological functions of DEPs. GO enrichment and KEGG pathway analyses were performed using the upregulated and downregulated DEPs, respectively. The top 10 GO biological process (BP) terms and KEGG pathways are shown in Fig. 2c, d.

The downregulated DEPs were mainly enriched in toll-like receptor 9, positive regulation of I-kappaB kinase/NF-kappaB, toll-like receptor, NIK/NF-kappaB, and regulation of I-kappaB kinase/NF-kappaB signaling pathway, and the upregulated DEPs were mainly enriched in regulation of adaptive immune response, regulation of lymphocyte mediated immunity, regulation of adaptive immune response based on somatic recombination of immune receptors built from immunoglobulin superfamily domains, regulation of leukocyte-mediated immunity, and adaptive immune response based on somatic recombination of immune receptors built from immunoglobulin superfamily domains signaling pathway in GO (biological process (BP)) terms, respectively (*P.adjust* < 0.05). The downregulated DEPs were mainly enriched in NF-kappa B, toxoplasmosis, arrhythmogenic right ventricular cardiomyopathy, ECM-receptor interaction, and hypertrophic cardiomyopathy signaling pathway, and the upregulated DEPs were mainly enriched in C-type lectin receptor, malaria, intestinal immune network for IgA production, graft-versus-host disease, and African trypanosomiasis signaling pathway in the KEGG pathway analyses, respectively (*P.adjust* < 0.05).

Several infection pathways were identified by KEGG pathway analysis in the present study, including toxoplasmosis, pathogenic Escherichia coli infection, Epstein-Barr virus infection, human immunodeficiency virus 1 infection, malaria, African trypanosomiasis, pertussis, amoebiasis, Yersinia infection, and tuberculosis (*P.adjust* < 0.05), highlighting an essential role of pathogenic infection in BD (Supplementary Figure S2a). The pathways of toxoplasmosis and malaria were significantly enriched using the upregulated and downregulated DEPs, respectively (Supplementary Figure S2b and c).

### Protein–protein interactions and correlations

To gain further insight into the protein–protein interactions between DEPs, correlation heatmaps and protein network interaction diagrams were constructed (Fig. 2e and Supplementary Figure S1c). Spearman’s correlation analysis showed that eukaryotic translation initiation factor 4 gamma 1 (EIF4G1) and (HEXIM P-TEFb complex subunit 1) HEXIM1 had the highest positive correlation (*r* = 0.93, *P* = 6.07e − 06), whereas tryptase alpha/beta 1 (TPSAB1) and IL10 had the highest negative correlation (*r* =  − 0.59, *P* = 2.14e − 22).

### Construction of the prediction model

**Fig. 3:**
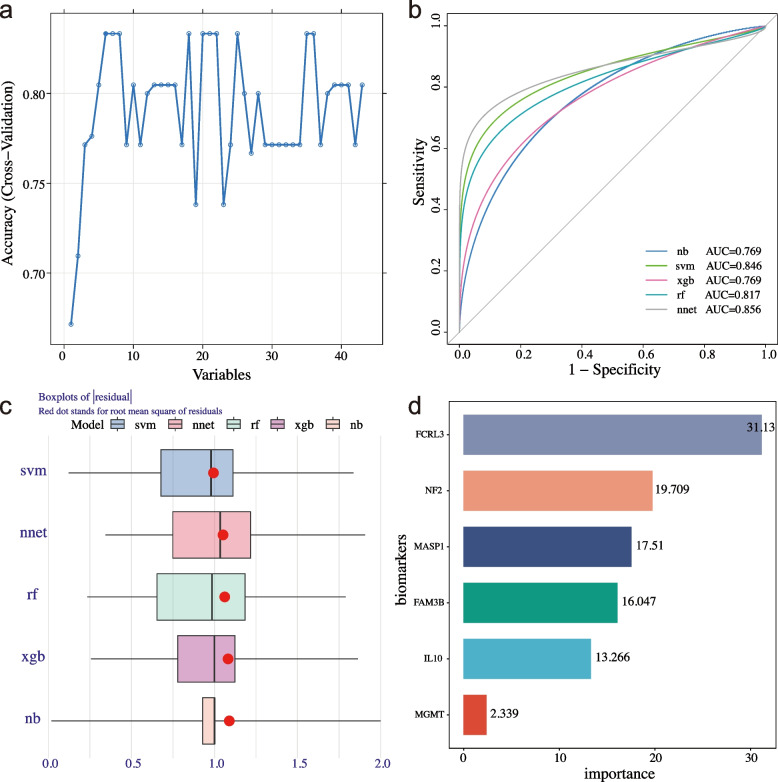
Construction of the prediction model. **A** The accuracy of the cross-validated RF model. **B** ROC curve and AUC value of NB, SVM, XGB, RF, and NNET models. **C** Boxplots of the residual distribution of NB, SVM, XGB, RF, and NNET models. **D** The importance of explanatory variables ranked by the SVM model

To identify potential biomarkers, five machine learning models were constructed using NB, SVM, XGB, RF, and NNET. First, a random forest algorithm was applied for the key feature selection. Six DEPs (IL10, FCRL3, MASP1, NF2, FAM3B, and MGMT) were selected as key features (Fig. 3a). NB, SVM, XGB, RF, and NNET models were constructed, and the prediction performance for BD was assessed using the AUC value and residual distribution. The results showed that the NNET and SVM models had the best performance (NNET, AUC value: 0.856, recall: 0.692, specificity: 0.857, precision: 0.900, accuracy:0.750, F1 score: 0.783; SVM, AUC value: 0.846, recall: 0.667, specificity: 0.750, precision: 0.800, accuracy: 0.700, F1 score: 0.727), followed by the RF model (RF, AUC value: 0.817, recall: 0.727, specificity: 0.778, precision: 0.800, accuracy: 0.750, F1 score: 0.762), whereas the NB and XGB models had the poorest and most unstable performance (Fig. 3b and Table 2). In addition, the SVM and NNET models had the lowest residual distribution compared to the other models (Fig. 3c). NNET model still had the best performance (AUC value: 0.941, recall: 0.684, specificity: 0.889, precision: 0.929, accuracy: 0.750, F1 score: 0.788) compared to the other models in the validation cohort (Supplementary Table S3).

**Table 2:** The performance of five machine learning models in the training cohort

	**Recall**	**Specificity**	**Precision**	**Accuracy**	**F1 score**	**AUC**
Naive Bayes	0.636	0.667	0.700	0.650	0.667	0.769
Support vector machine	0.667	0.750	0.800	0.700	0.727	0.846
Extreme gradient boosting	0.700	0.700	0.700	0.700	0.700	0.769
Random forest	0.727	0.778	0.800	0.750	0.762	0.817
Neural network	0.692	0.857	0.900	0.750	0.783	0.856

Based on these results, the NNET model exhibited the best prediction performance. The explanatory variables were ranked according to their importance (Fig. 3d). Besides, we further validated the biomarkers expression of IL10, FCRL3, MASP1, NF2, FAM3B, and MGMT in the validation cohort (Supplementary Figure S3). The results in the validation cohort were consistent with those in the training cohort. In addition, the correlation between these potential biomarkers and disease activity or disease duration was performed in our study, but no significant correlations were identified. The results were shown in (Supplementary Figure S4 and S5).

To further investigate the potential effects of treatments on plasma proteins, the protein expression level was compared between treated (glucocorticoids (GCs) or other immunosuppressive agents) and non-treated groups in BD patients. Four proteins were identified to be weak different between groups (Supplementary Figure S6a and b and Supplementary Table S4). Overall, the effect of treatments on the protein expression levels was very weak.

### Consensus clustering analysis in BD

**Fig. 4:**
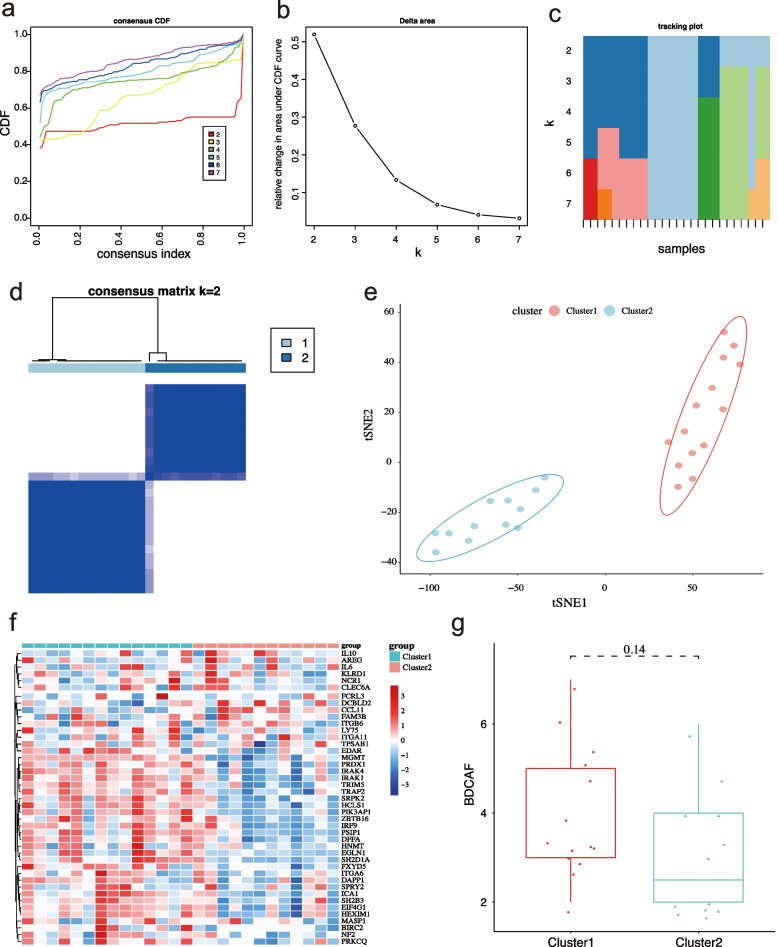
Consensus clustering of BD patients. **A** The consensus clustering CDF curve for *K* = 2–7. **B** The delta area score of the CDF curve for *k* = 2–7. **C** Tracking plot for *k* = 2–7 in BD patients. **D** Consensus clustering was the most stable when *K* = 2. **E** PCA based on 43 DEPs, different clinical phenotype, sex, age. **F** Heatmap showing the expression of 43 DEPs between the cluster 1 and cluster 2 groups. **G** Comparison of the score of BDCAF between the cluster 1 and cluster 2 groups

We constructed consensus clusters of BD patients based on the 43 DEPs, clinical phenotypes, sex, and age. The results of the cumulative distribution function (CDF) curves (Fig. 4a) and the relative change in area under for the CDF curve (*K* = 2–7) (Fig. 4b) demonstrated that consensus clustering was the most stable when *K* = 2 (Fig. 4c). Patients with BD were divided into two distinct clusters, cluster 1 and cluster 2 (Fig. 4d, e). We also plotted a clustering heatmap to present the expression levels of immune response-related proteins in both BD patient subsets (Fig. 4f). Protein expression levels were compared between cluster 1 and cluster 2. There were 21 upregulated proteins and one downregulated protein in cluster 1 compared to cluster 2 (Table 3).

**Table 3:** Comparison of immune-related proteins profile between cluster 1 and cluster 2

	**Name**	**Cluster 1 (*n* = 14)**	**Cluster 2 (*n* = 12)**	***P.value***
1	TRIM5	3.18 (2.98–3.55)	2.31 (1.58–2.54)	2.88E − 05
2	SH2D1A	3.06 (1.93–3.64)	0.67 (0.41–1.33)	0.000139992
3	PIK3AP1	4.38 (4.18–4.71)	2.74 (2.48–3.12)	0.000244158
4	HCLS1	5.20 (4.93–5.41)	4.29 (3.80–4.62)	0.000318502
5	DFFA	5.55(5.21–6.19)	4.29 (4.04–5.00)	0.000527869
6	TRAF2	4.13 (3.82–4.65)	2.93 (2.46–3.47)	0.000671796
7	PRDX1	3.15 (2.91–3.42)	2.56 (2.18–2.92)	0.001067128
8	PSIP1	3.76 (2.45–4.37)	1.42 (1.02–2.30)	0.001332823
9	IRAK1	2.48 (2.13–2.62)	1.61 (1.26–2.16)	0.001653189
10	SRPK2	2.81 (2.33–3.21)	1.76 (0.59–2.02)	0.001653189
11	MGMT	7.30 (7.28–7.33)	6.38 (6.05–6.97)	0.00204003
12	ICA1	1.63 (1.33–2.04)	1.06 (0.76–1.33)	0.00204003
13	IRF9	2.28 (1.94–2.49)	1.06 (0.78–1.85)	0.00305435
14	IRAK4	4.41 (4.13–4.69)	3.55 (2.86–4.15)	0.004480156
15	SH2B3	4.71 (4.56–4.89)	4.05 (3.65–4.57)	0.004480156
16	CCL11	6.71 (6.40–6.97)	7.33 (7.10–7.64)	0.007681332
17	EGLN1	1.57 (0.40–2.68)	0.39 (0.16–0.57)	0.007681332
18	PRKCQ	1.30 (1.16–1.89)	0.92 (0.73–1.18)	0.009380022
19	ZBTB16	3.27 (2.59–3.75)	1.94 (1.20–2.49)	0.010766539
20	HEXIM1	5.89 (5.51–6.30)	4.85 (4.54–5.94)	0.017334562
21	FXYD5	1.03 (0.87–1.34)	0.85 (0.65–0.90)	0.032769617
22	EDAR	2.71 (2.23–3.42)	2.20 (1.61–2.51)	0.040689398

### The demographic and clinical features between two subsets of BD patients

We compared the differences in protein expression levels and clinical features between cluster 1 and cluster 2. In addition, to assess the scores BDCAF, patients were asked about the presence of clinical symptoms over the 4 weeks prior to when they visited us. Clinical features and BDCAF scores are shown in Table 4. A total of 14 BD patients were grouped into cluster 1, while 12 BD patients were grouped into cluster 2. The mean age of cluster 1 and cluster 2 patients were 22.57 ± 7.70 years and 45.25 ± 9.84 years, respectively. A significant difference in age distribution was observed between the two subsets (*P* < 0.001). Although the patients from cluster 1 had higher BDCAF scores (3.93 ± 1.44 versus (vs). 3.08 ± 1.38)), this difference was not significant. Furthermore, there was no significant difference in the clinical features between the two groups. A higher frequency of mouth ulcers (13 (92.9%) vs. 6 (50.0%)), and genital ulcers (10 (71.4%) vs. 4 (33.3%)) was observed for cluster 1. There were no statistical differences in the use of immunosuppression between the two groups. Besides, a significant difference *(P* < 0.05) was found in terms of disease duration between cluster 1 (24.0 (17.25–54.50)) and cluster 2 (80.0 (36.0–215.2)). We further investigated the correlation between the expression level of DEPs and disease duration. Pearson correlation tests revealed that the expression level of TRIM5, Egl-9 family hypoxia inducible factor 1 (EGLN1), SH2D1A, and DFFA were positive correlation with disease duration (*P* < 0.05) (Supplementary Figure S7) and age positively correlated with disease duration (*r* = 0.42, *P* = 0.032).

**Table 4:** Scores of Behcet’s Disease Current Activity Form (BDCAF)

	**Overall**	**Cluster 1**	**Cluster 2**	***p***
*n*	26	14	12	
Sex (%)				
Female	13 (50.0)	5 (35.7)	8 (66.7)	0.238
Male	13 (50.0)	9 (64.3)	4 (33.3)	
Age (mean (SD))	33.04 (14.37)	22.57 (7.70)	45.25 (9.84)	** < 0.001**
Disease duration (months) (median (Q1, Q3))	37.0 (24.0–75.0)	24.0 (17.25–54.50)	80.0 (36.0–215.2)	**0.022**
Treatment (yes, %)	7 (26.90)	4 (28.6%)	3 (25.0)	1.000
Headaches (%)	7 (26.9)	5 (35.7)	2 (16.7)	0.517
Mouth ulcers (%)	19 (73.1)	13 (92.9)	6 (50.0)	0.044
Genital ulcers (%)	14 (53.8)	10 (71.4)	4 (33.3)	0.122
Erythema nodosum (%)	11 (42.3)	7 (50.0)	4 (33.3)	0.646
Pustules (%)	7 (26.9)	5 (35.7)	2 (16.7)	0.517
Arthralgia (%)	5 (19.2)	2 (14.3)	3 (25.0)	0.848
Arthritis (%)	4 (15.4)	1 (7.1)	3 (25.0)	0.476
Nausea/vomiting (%)	6 (23.1)	3 (21.4)	3 (25.0)	1.000
Diarrhea (%)	1 (3.8)	1 (7.1)	0 (0.0)	1.000
Any eye problems (%)	14 (53.8)	6 (42.9)	8 (66.7)	0.413
Any new CNS activity (%)	2 (7.7)	1 (7.1)	1 (8.3)	1.000
Any new major vascular activity (%)	2 (7.7)	1 (7.1)	1 (8.3)	1.000
BDCAF (mean (SD))	3.54 (1.45)	3.93 (1.44)	3.08 (1.38)	0.141

Treatment: GCs or other immunosuppressive agents

### DEPs between Behcet’s disease with and without uveitis

To investigate whether there are any differences between Behcet’s disease with and without uveitis, we compared the protein expression levels (Supplementary Table S5). The expression of PLXNA4 was significantly differentially expressed between BD with uveitis (BDU) and that without uveitis (BDNU) in the training cohort (*P* = 0.0054) and in the validation cohort (*P* = 0.0008) (Supplementary Figure S8).

## Discussion

BD is a chronic, multisystem autoinflammatory disorder. The diagnosis of BD mainly relies on clinical symptoms. In this study, we examined the expression levels of immune response-related proteins in the plasma of patients with BD using the Olink Immune Response panel. The results demonstrated aberrant expression of immune response-related proteins profiles in BD patients. Potential biomarkers were identified by constructing predictive models using machine learning algorithms. We also constructed a novel molecular disease classification model to identify the subsets of BD.

The etiology of BD remains unknown. We measured the expression levels of immune response-related protein to investigate the immunopathogenesis of BD. A total of 43 DEPs were identified in the BD and HC groups. The results of GO and KEGG enrichment analyses highlighted that the NF-κB signaling pathway and Toll like receptor 9 (TLR9) signaling pathway are involved in the occurrence of BD. These results are consistent with those of a previous study. Verrou et al. performed RNA-sequencing analysis in peripheral blood mononuclear cells and found that the NF-κB signaling pathway is related to BD [27]. Previous studies also reported that the NF-κB signaling pathway could protect T cells against CD95-mediated apoptosis in BD [28]. The NF-κB signaling pathway is considered a typical pro-inflammatory pathway, and the activation of signaling pathways induces the production of various proinflammatory cytokines such as IL-6 and IL-8 [29]. The NF-κB signaling pathway is also involved in the development of other rheumatic autoimmune diseases [30]. Activation of the NF-κB signaling pathway induces chronic inflammation of the synovium in rheumatoid arthritis [31].

TLR9 signaling pathway is essential for the regulation of both innate and adaptive immunity, and it is also involved in the production of type I interferons (IFNs) [32]. A recent study reported that dysregulation of TLR9 contributes to the production of IFN-γ and leads to fatal inflammatory disease in neonates [33]. Activation of the TLR9 signaling pathway has been observed in patients with primary Sjögren’s syndrome based on single cell phosphorylation profiling [34]. Additionally, in an experimental autoimmune encephalomyelitis (EAE) animal model of multiple sclerosis, pathogens have been attributed to TLR9-mediated innate immunity [35]. Overall, our study further identified the activation of the NF-κB and TLR9 signaling pathways in the plasma of BD. Together, these studies indicate that NF-κB and TLR9 signaling pathways are involved in the immunopathogenesis of BD. The DEPs were also enriched in infections-related signaling pathways such as toxoplasmosis and Epstein-Barr virus infection. Although the role of toxoplasmosis and Epstein-Barr virus infection in the pathogenesis of BD is less well understood, some studies have demonstrated that toxoplasmosis and Epstein-Barr virus infection are the risk factors for other systemic immune diseases, including rheumatoid arthritis and systemic lupus erythematosus [36, 37], and were associated with the activation of the NF-κB signaling pathway [38, 39]. In addition, a recent plasma proteomic study in BD patients also revealed that several infection pathways, for example, pertussis, amoebiasis, and tuberculosis, were associated with the pathogenesis of BD [40]. These pathways implicated the role of infection in the pathogenesis of BD.

IL-10, FCRL3, MASP1, NF2, FAM3B, and MGMT are potential candidate biomarkers for BD. The diagnosis of BD was made based on clinical symptoms [41]. To the best of our knowledge, the current study is the first to use machine learning algorithms to identify the potential candidate biomarkers in BD.

IL-10 is an anti-inflammatory cytokine that can inhibit Th1 cytokine production and Th1 cell differentiation [6]. Our results are consistent with the results reported by Aridogan et al., which described the elevated level of IL-10 in the serum of active BD [42]. In addition, our previous study assessed the aqueous cytokine levels in BD and senile cataract patients. However, the expression level of IL-10 was not statistically significant, which might be because the intraocular inflammations of BD were in the inactive phase [43]. Overall, the overexpression of IL 10 may represent a compensatory mechanism in response to chronic inflammation in BD. The overexpression of IL 10 may play an important role in dampening excessive inflammation by inhibiting IL 6, which is also highly expressed in our study [44]. Another possible explanation is that IL-10 may have a dual role in immune responses. While IL 10 is generally considered to be anti-inflammatory, it can also promote inflammation under certain circumstances [45]. For example, IL 10 has been shown to enhance the inflammatory response in some autoimmune diseases, such as systemic lupus erythematosus (SLE) [46]. It is possible that IL 10 has a similar pro-inflammatory effect in BD.

FCRL3 is an orphan receptor, which is only expressed on the lymphocyte cell surface. it can inhibit the secretion of TNF-α, IL 1β, IL 6, and IL-8 by promoting the expression of IL 10 in multiple sclerosis [47]. In addition, a single nucleotide polymorphism in the FCRL3 promoter region binding of the NF-κB is associated with rheumatoid arthritis, autoimmune thyroid disease, and systemic lupus erythematosus [48]. Our previous study also found associations between a single nucleotide polymorphism of FCRL3 and BD susceptibility in the Chinese population [49]. MASP1 is a serine protease involved in complement system. It is essential for defense against invading pathogens and altering host structures [50]. NF2, FAM3B, and MGMT are primarily involved in regulating the tumor immune microenvironment [51–53]. We reported, for the first time, a significant difference in the expression levels of NF2, FAM3B, and MGMT between BD and HC. However, further experiments are needed to explore the functional role of NF2, FAM3B, and MGMT in BD and other autoimmune diseases.

We report a novel molecular disease classification model for BD based on an unsupervised consensus clustering algorithm. DEPs, clinical phenotypes, sex, and age were used to construct the model. BD patients were divided into two subsets, cluster 1 with 14 patients and cluster 2 with 12 patients, characterized by distinct cytokine production profiles and disease activity. The characterization of cluster 1 was high disease activity and high TRIM5, SH2D1A, PIK3AP1, HCLS1, and DFFA expression. The characterization of cluster 2 showed low disease activity associated with a higher expression of CCL11. Our molecular disease model differed from the previous clinical classification model in that it is a novel immunophenotype for BD [54]. This model provides insight into the immunopathogenesis of BD and might help further refine the classification and diagnosis of BD. Besides, TRIM5, EGLN1, SH2D1A, and DFFA were correlated with disease duration, which may explain the classification model.

Another interesting finding from our study was that PLXNA4 (plexin A4) is a DEP between BDU and BDNU, whereby PLXNA4 expression was down-regulated in BDU. A previous study indicated that cytokines could impair vascular integrity by downregulating the expression of PLXNA4 [55]. This may explain the occurrence of retinal vasculitis in BDU.

Our study had some limitations. Most patients in our study previously received small doses of immunosuppressants; however, the effect of treatments was weak. Further clinical significance and function of candidate biomarkers need to be comprehensively investigated. It is undeniable that sample sizes are small in our study. We only compared the uveitis phenotype and without uveitis phenotype in BD patients. Further expanded experimental sample size and analysis of the relationship between immune response-related proteins and other phenotypes of BD patients will be necessary.

## Conclusions

In summary, our results revealed that immune response-related proteins were differentially and significantly expressed in the plasma of patients with BD compared with HC. Based on machine learning algorithms, we confirmed that IL-10, FCRL3, MASP1, NF2, FAM3B, and MGMT may be promising biomarkers for BD. Furthermore, PLXNA4 may be a valuable biomarker to predict the occurrence of uveitis in patients with BD. A novel disease classification model using proteomic and clinical data has been constructed to identify subsets of patients with BD. However, further prospective studies are required to validate these findings.

## Supplementary Information

**Additional file 1: Supplementary Figure S1.** (a) The quality control of the sample. The red color represents the failure of quality control. (b) Volcano plot of DEPs (c) The protein-protein interaction (PPI) network of 43 DEPs.

**Additional file 2: Supplementary Figure S2.** Enriched KEGG pathways associated with infection.

**Additional file 3: Supplementary Figure S3.** Comparison of the expression of the biomarkers between BD and HC in the validation cohort.

**Additional file 4: Supplementary Figure S4.** The correlation between biomarkers and disease activity.

**Additional file 5: Supplementary Figure S5.** The correlation between biomarkers and disease duration.

**Additional file 6: Supplementary Figure S6.** Comparison of the expression level between treated and non-treated groups in BD patients.

**Additional file 7: Supplementary Figure S7.** The correlation between the expression level of proteins and disease duration.

**Additional file 8: Supplementary Figure S8.** Comparison of the expression level of PLXNA4 between BDU and BDNU in the validation cohort.

**Additional file 9: Supplementary Table S1.** Clinical features of 27 Behcet’s disease (BD) patients

**Additional file 10: Supplementary Table S2.** Comparison of immune-related proteins profile between BD and HC.

**Additional file 11: Supplementary Table S3.** The performance of five machine learning models in the training cohort.

**Additional file 12: Supplementary Table 4.** Comparison of immune-related proteins expression level between treated and non-treated groups in BD patients.

**Additional file 13: Supplementary Table S5.** Comparison of the differential expression of immune- related proteins between Behcet’s disease (BDU) with and without uveitis (BDNU).

## Data Availability

Supplementary data are available at Arthritis Research & Therapy online.

## Abbreviations

AUC: Area under the curve
BD: Behcet’s disease
BDCAF: Behçet Disease Current Activity Form
BDNU: Behcet’s disease without uveitis
BDU: Behcet’s disease with uveitis
BP: Biological process
CCL11: C-C motif chemokine ligand 11
CV: Coefficient of variance
DEPs: Differentially expressed proteins
DFFA: DNA fragmentation factor subunit alpha
FAM3B: FAM3 metabolism regulating signaling molecule B
FCRL3: Fc receptor like 3
GCs: Glucocorticoids
GO: Gene ontology
HC: Healthy controls
HCLS: Hematopoietic cell-specific Lyn substrate 1
IL10: Interleukin 10
IL6: Interleukin 6
KEGG: Kyoto Encyclopedia of Genes and Genomes
MASP: Mannan-binding lectin serine peptidase 1
MGMT: O-6-Methylguanine-DNA methyltransferase
NB: Naive Bayes
NF2: NF2, moesin-ezrin-radixin like (MERLIN) tumor suppressor
NF-ΚB: Nuclear factor-κB
NNET: Neural network
NPX: Normalized protein expression
PCA: Principal component analysis
PEA: Proximity extension assay
PIK3AP1: Phosphoinositide-3-kinase adaptor protein 1
PLXNA4: Plexin A4
PPI: Protein network interaction
RF: Random forest
ROC: Receiver operating characteristic
SH2D1A: SH2 domain-containing 1A
SVM: Support vector machine
TH17: T helper 17
TRIM21: Tripartite motif-containing 21
TRIM5: Tripartite motif-containing 5
tsne: T-distributed stochastic neighbor embedding
VS: Versus
XGB: Extreme gradient boosting
