# Supplementary figures and images for: Identification of the immune-related biomarkers in Behcet’s disease by plasma proteomic analysis
Source: Arthritis Res Ther. 2023 Jun 1;25:92. doi: 10.1186/s13075-023-03074-y (PMC10233985; doi:10.1186/s13075-023-03074-y)

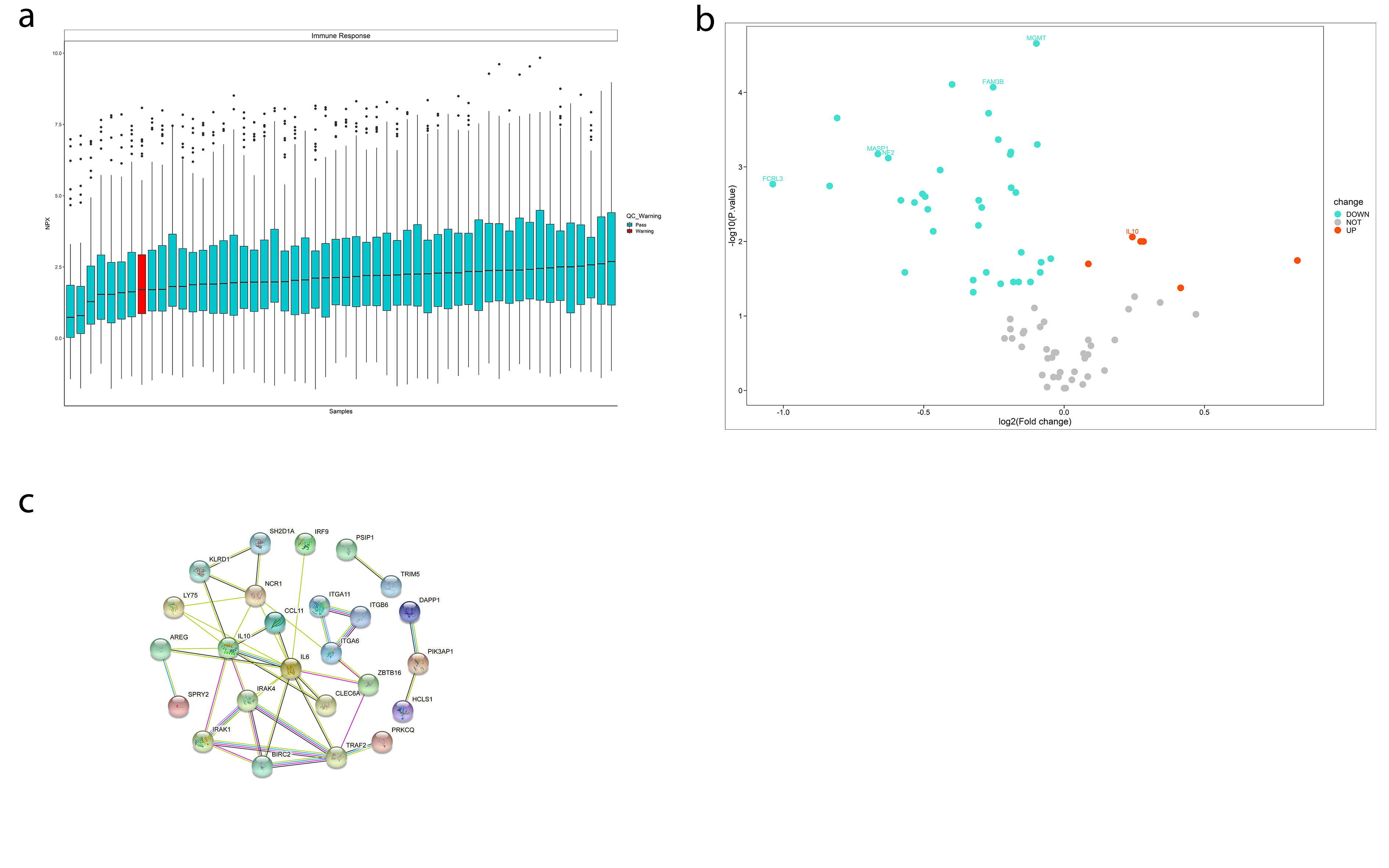

Supplement: Supplementary file 1 — Additional file 1: Supplementary Figure S1. (a) The quality control of the sample. The red color represents the failure of quality control. (b) Volcano plot of DEPs (c) The protein-protein interaction (PPI) network of 43 DEPs. [file 13075_2023_3074_MOESM1_ESM.tif]

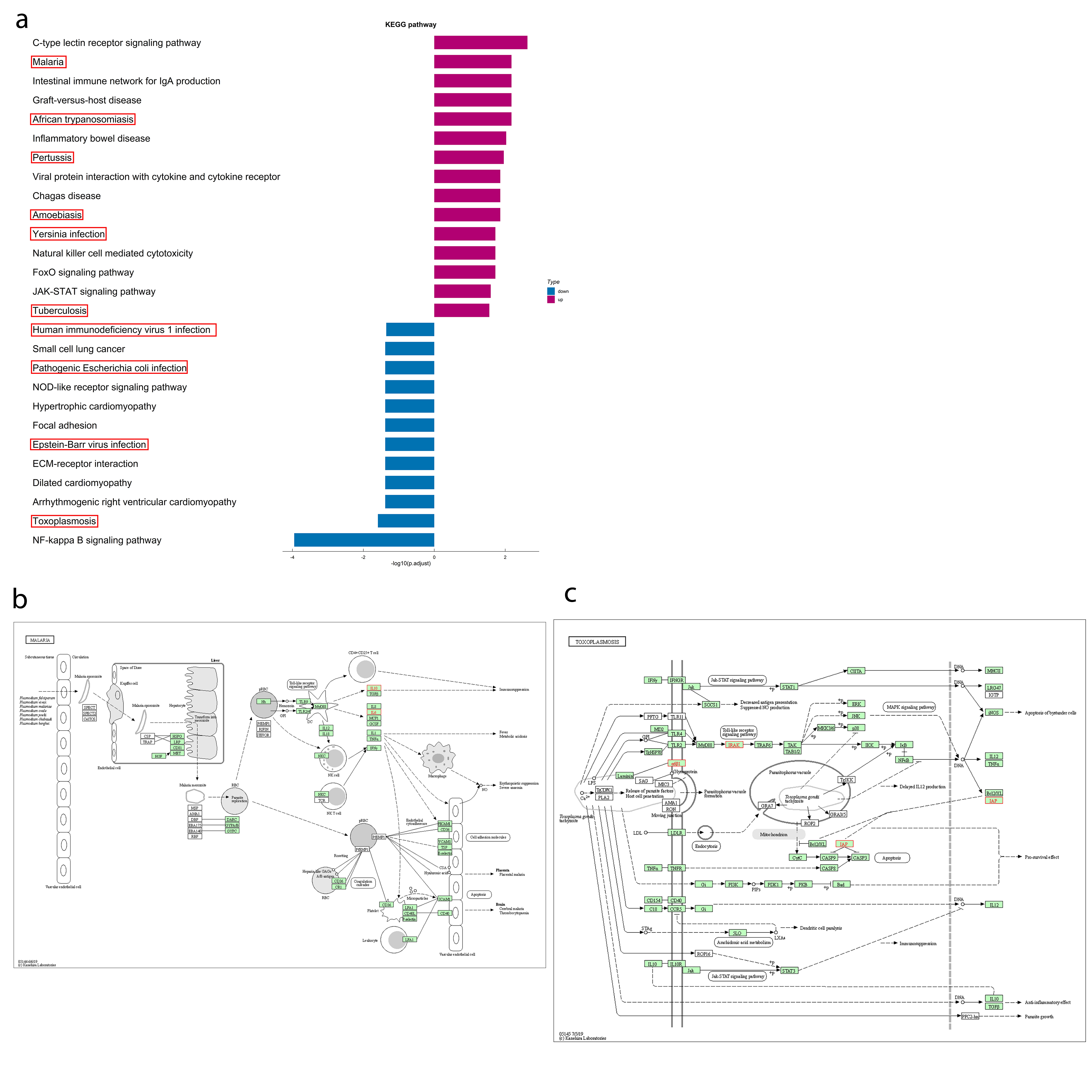

Supplement: Supplementary file 2 — Additional file 2: Supplementary Figure S2. Enriched KEGG pathways associated with infection. [file 13075_2023_3074_MOESM2_ESM.tif]

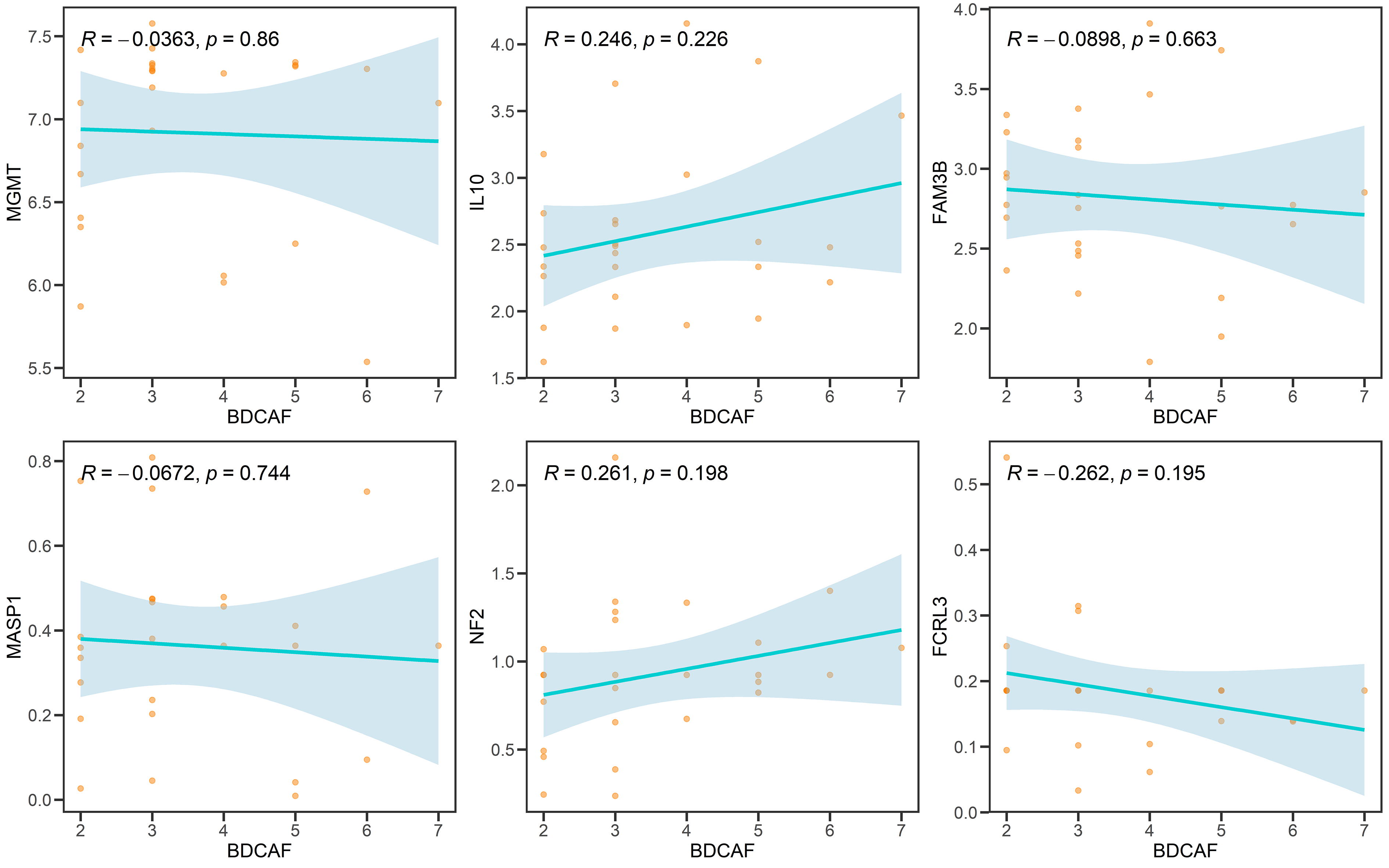

Supplement: Supplementary file 4 — Additional file 4: Supplementary Figure S4. The correlation between biomarkers and disease activity. [file 13075_2023_3074_MOESM4_ESM.tif]

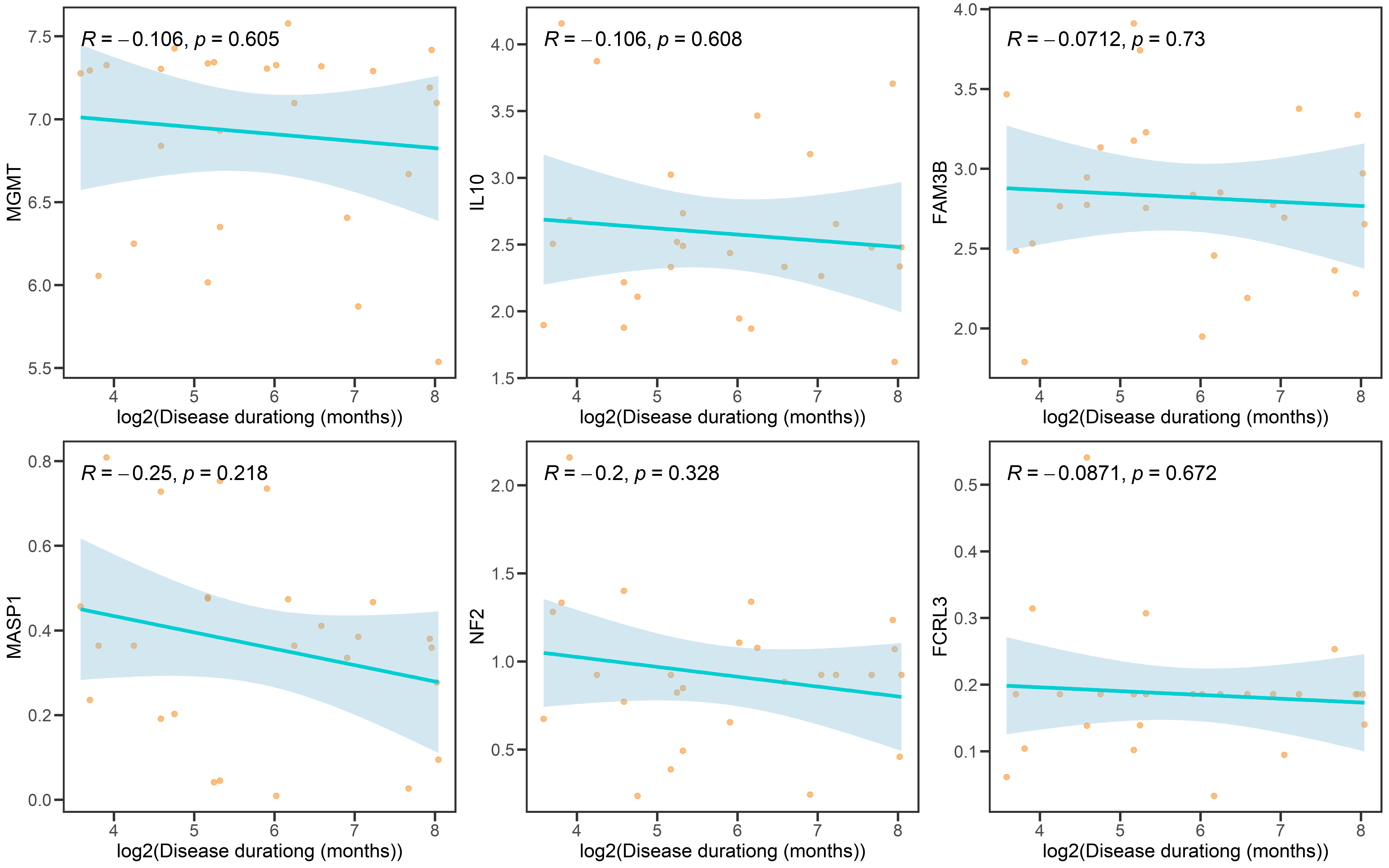

Supplement: Supplementary file 5 — Additional file 5: Supplementary Figure S5. The correlation between biomarkers and disease duration. [file 13075_2023_3074_MOESM5_ESM.tif]

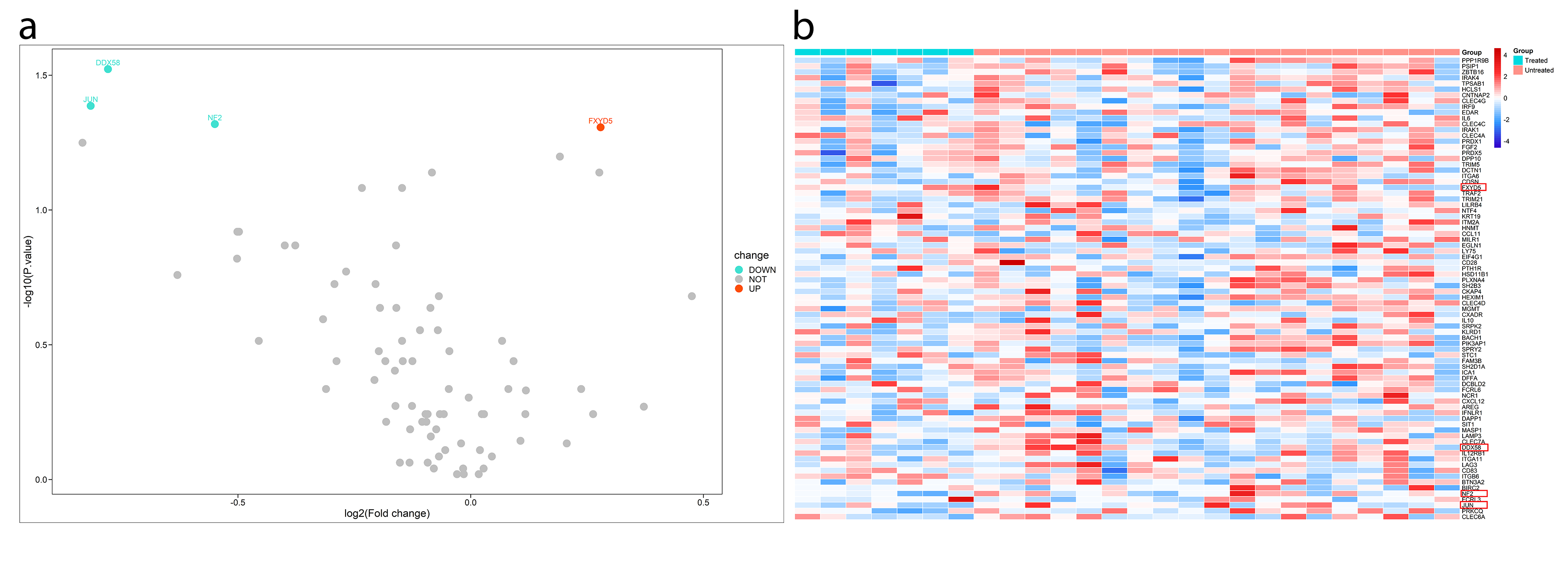

Supplement: Supplementary file 6 — Additional file 6: Supplementary Figure S6. Comparison of the expression level between treated and non-treated groups in BD patients. [file 13075_2023_3074_MOESM6_ESM.tif]

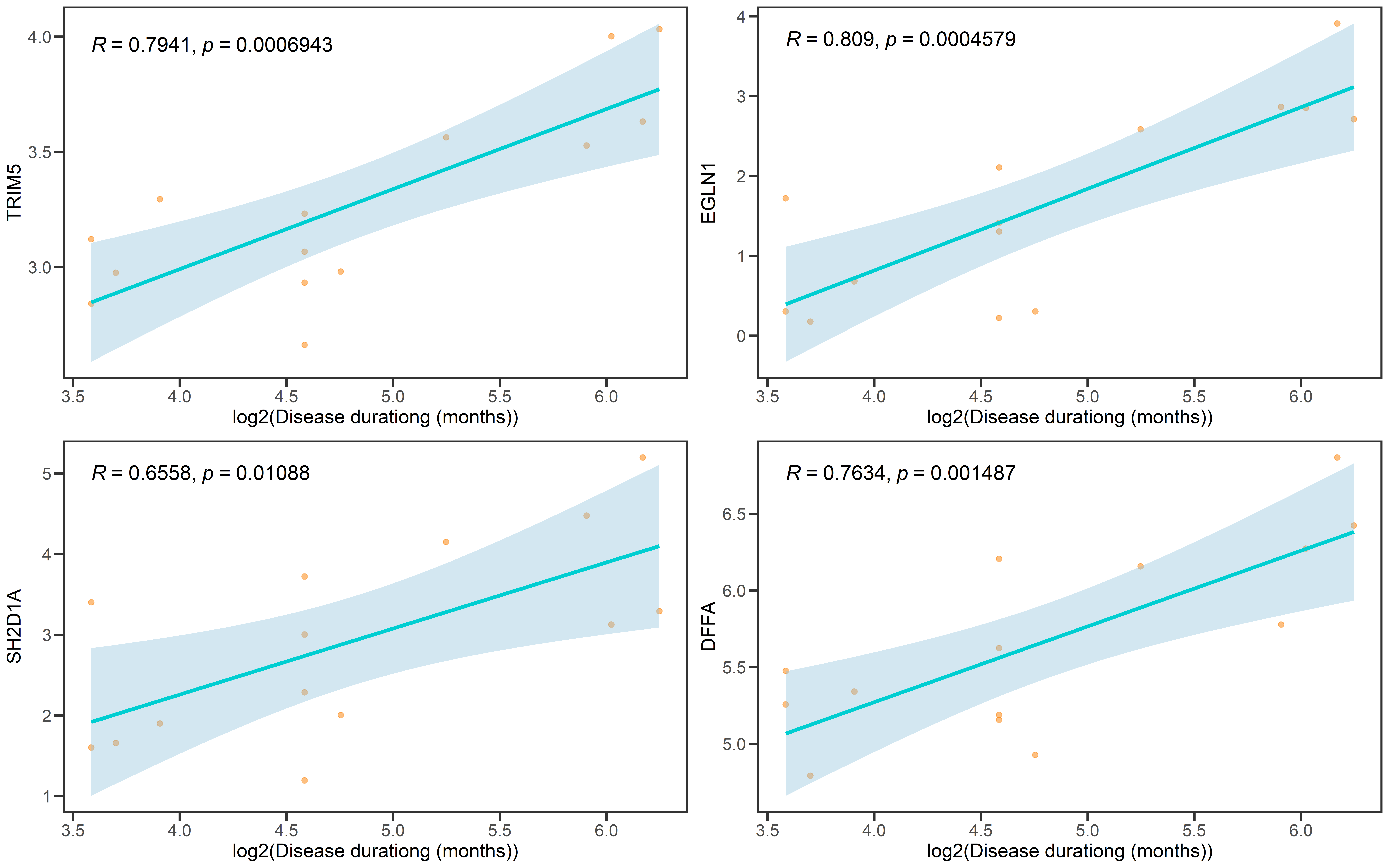

Supplement: Supplementary file 7 — Additional file 7: Supplementary Figure S7. The correlation between the expression level of proteins and disease duration. [file 13075_2023_3074_MOESM7_ESM.tif]
