# Supplementary material for: Identification of the immune-related biomarkers in Behcet’s disease by plasma proteomic analysis
Source: Arthritis Res Ther. 2023 Jun 1;25:92. doi: 10.1186/s13075-023-03074-y (PMC10233985; doi:10.1186/s13075-023-03074-y)
Supplement: Supplementary file 9 — Additional file 9: Supplementary Table S1. Clinical features of 27 Behcet’s disease (BD) patients [file 13075_2023_3074_MOESM9_ESM.docx]

**Supplementary Table S1** Clinical features of 27 Behcet’s disease (BD) patients

| **Patient NO.** | **Sex** | **Age** | **Oral or Genital ulcers** | **Skin** | **Joint** | **Uveitis** | **Vascular** | **Neurological** | **Gastrointestinal** | **Disease duration**  **(months)** | **Treat strategy** |
| --- | --- | --- | --- | --- | --- | --- | --- | --- | --- | --- | --- |
| BD1 | M | 43 | 1 | 0 | 0 | 1 | 1 | 0 | 0 | 249 | NA |
| BD2 | F | 49 | 1 | 0 | 0 | 1 | 0 | 0 | 0 | 36 | NA |
| BD3 | F | 60 | 1 | 0 | 0 | 1 | 0 | 0 | 0 | 40 | NA |
| BD4 | M | 34 | 1 | 0 | 0 | 1 | 1 | 0 | 0 | 19 | Colchicine  0.25mg bid |
| BD5 | M | 18 | 1 | 0 | 0 | 1 | 0 | 0 | 0 | 96 | GCs 5mg qd;  Thalidomide  25mg qd |
| BD6 | F | 16 | 1 | 0 | 0 | 1 | 0 | 0 | 0 | 24 | NA |
| BD7 | M | 42 | 1 | 1 | 1 | 1 | 0 | 0 | 0 | 264 | GCs 10mg qd;  Thalidomide  50mg tid |
| BD8 | M | 20 | 1 | 0 | 0 | 1 | 0 | 0 | 0 | 65 | NA |
| BD9 | F | 17 | 1 | 1 | 0 | 1 | 0 | 0 | 0 | 12 | NA |
| BD10 | F | 62 | 1 | 0 | 0 | 1 | 0 | 0 | 0 | 14 | NA |
| BD11 | M | 31 | 1 | 0 | 0 | 1 | 0 | 0 | 0 | 38 | GCs 5mg bid;  MTX 15mg qw |
| BD12 | M | 29 | 1 | 1 | 0 | 0 | 0 | 0 | 0 | 72 | NA |
| BD13 | F | 55 | 1 | 0 | 0 | 0 | 0 | 0 | 0 | 260 | NA |
| BD14 | F | 37 | 1 | 0 | 1 | 0 | 0 | 0 | 0 | 132 | NA |
| BD15 | F | 24 | 1 | 1 | 0 | 0 | 0 | 0 | 0 | 245 | NA |
| BD16 | M | 42 | 1 | 1 | 1 | 0 | 0 | 0 | 0 | 204 | NA |
| BD17 | F | 41 | 1 | 1 | 1 | 0 | 0 | 1 | 1 | 120 | GCs 40mg qd;  MTX 15mg qw |
| BD18 | M | 40 | 1 | 0 | 1 | 0 | 0 | 0 | 0 | 12 | Thalidomide  25mg qd |
| BD19 | F | 17 | 1 | 0 | 1 | 0 | 0 | 1 | 0 | 13 | NA |
| BD20 | M | 31 | 1 | 0 | 0 | 0 | 0 | 0 | 1 | 15 | NA |
| BD21 | M | 17 | 1 | 0 | 0 | 0 | 0 | 0 | 0 | 27 | GCs 10mg bid；  Thalidomide  100mg qd |
| BD22 | M | 25 | 1 | 0 | 0 | 0 | 1 | 0 | 0 | 60 | NA |
| BD23 | M | 31 | 1 | 1 | 0 | 0 | 1 | 0 | 0 | 76 | NA |
| BD24 | F | 13 | 1 | 0 | 0 | 0 | 0 | 0 | 0 | 24 | Colchicine  0.50mg qd |
| BD25 | M | 18 | 1 | 0 | 0 | 1 | 0 | 0 | 0 | 150 | NA |
| BD26 | F | 41 | 1 | 0 | 1 | 1 | 0 | 0 | 0 | 40 | NA |
| BD27 | F | 48 | 1 | 1 | 0 | 1 | 0 | 0 | 0 | 36 | NA |

F: female; M: male; 1 = involvement; 0 = not involvement; NA: not applicable; GCs: glucocorticoids; MTX: methotrexate; qd: once daily; bid: twice daily; tid: three times daily; qw: once per week
