# Supplementary material for: Identification of the immune-related biomarkers in Behcet’s disease by plasma proteomic analysis
Source: Arthritis Res Ther. 2023 Jun 1;25:92. doi: 10.1186/s13075-023-03074-y (PMC10233985; doi:10.1186/s13075-023-03074-y)
Supplement: Supplementary file 10 — Additional file 10: Supplementary Table S2. Comparison of immune-related proteins profile between BD and HC. [file 13075_2023_3074_MOESM10_ESM.docx]

**Supplementary Table S2.** Comparison of immune-related proteins profile between BD and HC.

|  | name | BD(n=26) | HC(n=25) | *p* |
| --- | --- | --- | --- | --- |
| 1 | MGMT | 7.23(6.47-7.32) | 7.46(7.34-7.59) | 2.20E-05 |
| 2 | IRAK1 | 2.15(1.63-2.5) | 2.78(2.45-2.99) | 7.80E-05 |
| 3 | FAM3B | 2.77(2.5-3.17) | 3.33(3.13-3.53) | 8.50E-05 |
| 4 | IRAK4 | 4.15(3.61-4.47) | 4.98(4.3-5.33) | 0.00019 |
| 5 | SH2D1A | 1.75(0.87-3.25) | 3.48(2.79-4.54) | 0.00022 |
| 6 | DFFA | 5.22(4.43-5.74) | 5.95(5.60-6.53) | 0.00043 |
| 7 | CCL11 | 7.00(6.63-7.35) | 7.45(7.28-7.71) | 5.00E-04 |
| 8 | HCLS1 | 4.81(4.28-5.24) | 5.41(5.19-5.72) | 0.00063 |
| 9 | MASP1 | 0.36(0.21-0.47) | 0.60(0.48-0.67) | 0.00067 |
| 10 | ITGB6 | 1.96(1.69-2.14) | 2.20(2.06-2.29) | 0.00068 |
| 11 | NF2 | 0.92(0.70-1.10) | 1.42(1.33-1.74) | 0.00076 |
| 12 | EDAR | 2.47(1.85-2.97) | 3.23(2.63-3.97) | 0.0011 |
| 13 | FCRL3 | 0.19(0.14-0.19) | 0.39(0.18-0.39) | 0.0017 |
| 14 | EGLN1 | 0.59(0.24-1.68) | 1.68(1.38-2.43) | 0.0018 |
| 15 | SH2B3 | 4.56(4.11-4.79) | 5.18(4.76-5.56) | 0.0019 |
| 16 | TPSAB1 | 4.78(4.37-5.43) | 5.39(5.22-5.61) | 0.0022 |
| 17 | SRPK2 | 2.22(1.70-2.81) | 2.99(2.44-3.75) | 0.0023 |
| 18 | ITGA6 | 1.09(0.72-1.39) | 1.61(1.17-1.90) | 0.0025 |
| 19 | PSIP1 | 2.54(1.39-4.05) | 4.07(3.37-4.97) | 0.0028 |
| 20 | TRAF2 | 3.77(2.97-4.36) | 4.65(3.79-4.94) | 0.0028 |
| 21 | FXYD5 | 0.88(0.81-1.11) | 1.32(1.03-1.70) | 0.0030 |
| 22 | TRIM5 | 2.89(2.37-3.28) | 3.48(3.15-3.86) | 0.0035 |
| 23 | ITGA11 | 0.91(0.64-1.14) | 1.19(1.01-1.53) | 0.0037 |
| 24 | PIK3AP1 | 3.74(2.68-4.39) | 4.49(4.07-5.25) | 0.0061 |
| 25 | PRKCQ | 1.18(0.84-1.43) | 1.62(1.44-1.88) | 0.0073 |
| 26 | IL10 | 2.48(2.23-2.72) | 2.10(2.00-2.35) | 0.0087 |
| 27 | NCR1 | 1.74(1.57-1.88) | 1.53(1.32-1.67) | 0.0100 |
| 28 | AREG | 2.48(2.30-3.35) | 2.16(2.07-2.44) | 0.0100 |
| 29 | DAPP1 | 5.64(4.74-5.94) | 5.97(5.49-6.59) | 0.0140 |
| 30 | DCBLD2 | 6.84(6.59-7.05) | 7.13(6.87-7.30) | 0.0170 |
| 31 | IL6 | 1.47(1.09-2.80) | 1.14(0.95-1.29) | 0.0180 |
| 32 | EIF4G1 | 7.14(6.31-7.31) | 7.37(7.00-7.60) | 0.0190 |
| 33 | KLRD1 | 5.94(5.52-6.15) | 5.5(5.26-5.74) | 0.0200 |
| 34 | ZBTB16 | 2.57(1.68-3.48) | 3.27(2.95-3.8) | 0.0260 |
| 35 | HNMT | 7.72(7.24-8.13) | 8.05(7.68-8.67) | 0.0260 |
| 36 | BIRC2 | 0.27(0.19-0.28) | 0.40(0.24-0.56) | 0.0260 |
| 37 | ICA1 | 1.36(1.12-1.76) | 1.81(1.38-2.12) | 0.0330 |
| 38 | PRDX1 | 2.96(2.58-3.22) | 3.19(2.86-3.56) | 0.0350 |
| 39 | LY75 | 1.14(1.01-1.32) | 1.28(1.17-1.59) | 0.0350 |
| 40 | HEXIM1 | 5.83(4.95-6.07) | 6.08(5.56-6.46) | 0.0350 |
| 41 | SPRY2 | 2.64(2.27-3.25) | 3.24(2.80-3.50) | 0.0370 |
| 42 | CLEC6A | 1.27(1.04-1.91) | 1.07(0.83-1.27) | 0.0420 |
| 43 | IRF9 | 1.97(1.17-2.36) | 2.24(1.97-2.54) | 0.0480 |

BD: Behcet’s disease. HC: Healthy controls. *p*: P value
