# Supplementary material for: Identification of the immune-related biomarkers in Behcet’s disease by plasma proteomic analysis
Source: Arthritis Res Ther. 2023 Jun 1;25:92. doi: 10.1186/s13075-023-03074-y (PMC10233985; doi:10.1186/s13075-023-03074-y)
Supplement: Supplementary file 11 — Additional file 11: Supplementary Table S3. The performance of five machine learning models in the training cohort. [file 13075_2023_3074_MOESM11_ESM.docx]

**Supplementary Table S3** The performance of five machine learning models in the training cohort.

|  | **Recall** | **Specificity** | **Precision** | **Accuracy** | **F1 Score** | **AUC** |
| --- | --- | --- | --- | --- | --- | --- |
| Naïve Bayes | 0.875 | 0.650 | 0.500 | 0.714 | 0.636 | 0.775 |
| Support Vector Machine | 0.000 | 0.481 | 0.000 | 0.464 | 0.000 | 0.592 |
| Extreme gradient boosting | 0.714 | 0.857 | 0.893 | 0.768 | 0.794 | 0.893 |
| Random Forest | 0.583 | 1.000 | 1.000 | 0.643 | 0.737 | 0.830 |
| Neural Network | 0.684 | 0.889 | 0.929 | 0.750 | 0.788 | 0.941 |
