# Supplementary material for: Identification of the immune-related biomarkers in Behcet’s disease by plasma proteomic analysis
Source: Arthritis Res Ther. 2023 Jun 1;25:92. doi: 10.1186/s13075-023-03074-y (PMC10233985; doi:10.1186/s13075-023-03074-y)
Supplement: Supplementary file 12 — Additional file 12: Supplementary Table 4. Comparison of immune-related proteins expression level between treated and non-treated groups in BD patients. [file 13075_2023_3074_MOESM12_ESM.docx]

**Supplementary Table4** Comparison of immune-related proteins expression level between treated and non-treated groups in BD patients.

|  | **BD with treatment**  **(n=7)** | **BD without treatment(n=19)** | **P value** |
| --- | --- | --- | --- |
| DDX58 | 1.19(0.97-1.93) | 2.69(1.58-3.29) | 0.030 |
| JUN | 0.27(0.23-0.59) | 0.72(0.50-0.74) | 0.041 |
| NF2 | 0.82(0.51-0.90) | 0.92(0.76-1.26) | 0.048 |
| FXYD5 | 0.99(0.94-1.20) | 0.86(0.64-1.00) | 0.049 |

n: sample sizes
