# Supplementary material for: Identification of the immune-related biomarkers in Behcet’s disease by plasma proteomic analysis
Source: Arthritis Res Ther. 2023 Jun 1;25:92. doi: 10.1186/s13075-023-03074-y (PMC10233985; doi:10.1186/s13075-023-03074-y)
Supplement: Supplementary file 13 — Additional file 13: Supplementary Table S5. Comparison of the differential expression of immune- related proteins between Behcet’s disease (BDU) with and without uveitis (BDNU). [file 13075_2023_3074_MOESM13_ESM.docx]

**Supplementary Table S5.** Comparison of the differential expression of immune- related proteins between Behcet’s disease (BDU) with and without uveitis (BDNU).

|  | BDU (n=14) | BDNU (n=12) | *P*-value |
| --- | --- | --- | --- |
| PLXNA4 | 5.55(5.27-5.85) | 6.33(5.87-6.85) | 0.0054 |
| CLEC7A | 2.41(2.08-2.52) | 2.04(1.84-2.30) | 0.0270 |
| PPP1R9B | 2.42(2.16-2.80) | 3.29(2.71-3.58) | 0.0407 |
| NTF4 | 0.62(0.43-0.70) | 0.74(0.66-0.97) | 0.0596 |
| CLEC6A | 1.49(1.12-2.12) | 1.10(0.75-1.50) | 0.0673 |
| EIF4G1 | 6.96(6.22-7.23) | 7.29(6.93-7.48) | 0.0849 |
| DCTN1 | 4.07(3.67-4.23) | 4.41(4.02-4.84) | 0.0950 |
| SH2B3 | 4.31(4.03-4.62) | 4.67(4.53-5.05) | 0.1060 |
| HEXIM1 | 5.44(4.82-5.93) | 6.00(5.40-6.33) | 0.1060 |
| DCBLD2 | 6.95(6.66-7.12) | 6.81(6.49-6.88) | 0.1060 |
| HNMT | 7.98(7.54-8.27) | 7.30(7.16-7.86) | 0.1179 |
| FCRL3 | 0.19(0.14-0.19) | 0.19(0.19-0.20) | 0.1306 |
| DDX58 | 2.77(1.81-3.35) | 1.63(1.20-2.77) | 0.1308 |
| CD83 | 1.32(1.16-1.58) | 1.11(0.95-1.33) | 0.1308 |
| JUN | 0.72(0.53-0.72) | 0.48(0.25-0.73) | 0.1388 |
| BACH1 | 2.43(1.58-2.67) | 2.77(2.32-2.98) | 0.1598 |
| ICA1 | 1.24(1.03-1.52) | 1.64(1.17-2.09) | 0.1598 |
| CXCL12 | 0.35(0.19-0.36) | 0.44(0.31-0.59) | 0.1703 |
| IRAK1 | 2.10(1.40-2.31) | 2.35(1.88-2.56) | 0.1932 |
| PRKCQ | 1.04(0.84-1.18) | 1.28(1.04-1.76) | 0.1984 |
| KLRD1 | 6.14(5.53-6.37) | 5.71(5.52-6.03) | 0.2116 |
| STC1 | 4.91(4.81-5.31) | 4.69(4.26-5.16) | 0.2116 |
| PRDX5 | 7.40(6.79-7.70) | 7.65(7.30-7.88) | 0.2312 |
| SRPK2 | 1.89(0.99-2.78) | 2.45(1.87-2.93) | 0.2520 |
| FAM3B | 2.98(2.68-3.37) | 2.73(2.48-2.88) | 0.2520 |
| ITGB6 | 2.01(1.94-2.14) | 1.78(1.58-2.07) | 0.2520 |
| TRIM5 | 2.67(1.86-3.20) | 2.98(2.64-3.35) | 0.2740 |
| CCL11 | 7.08(6.77-7.66) | 6.93(6.54-7.17) | 0.3217 |
| TRAF2 | 3.33(2.76-4.27) | 3.87(3.36-4.39) | 0.3474 |
| CLEC4A | 2.71(2.44-2.99) | 2.50(2.25-2.82) | 0.3744 |
| IFNLR1 | 1.71(1.51-1.91) | 1.57(1.25-1.82) | 0.4025 |
| LAG3 | 1.56(1.29-2.17) | 1.38(1.25-1.58) | 0.4025 |
| BTN3A2 | 1.76(1.49-1.98) | 1.90(1.72-2.12) | 0.4025 |
| BIRC2 | 0.27(0.24-0.27) | 0.27(0.17-0.39) | 0.4071 |
| PIK3AP1 | 3.19(2.68-4.25) | 4.17(2.69-4.61) | 0.4319 |
| CLEC4C | 2.87(2.06-3.22) | 3.08(2.52-3.55) | 0.4624 |
| CXADR | 1.19(1.03-1.42) | 1.09(0.93-1.16) | 0.4624 |
| FCRL6 | 2.51(2.06-3.13) | 2.37(2.16-2.66) | 0.4624 |
| SPRY2 | 2.59(2.29-2.85) | 2.79(2.29-3.69) | 0.4940 |
| LAMP3 | 3.38(2.90-4.55) | 3.26(3.05-3.37) | 0.4940 |
| IL12RB1 | 0.92(0.64-1.16) | 0.76(0.63-1.05) | 0.4940 |
| ITGA6 | 1.09(0.62-1.28) | 1.15(0.85-1.44) | 0.5267 |
| EGLN1 | 0.87(0.38-1.68) | 0.43(0.19-1.66) | 0.5267 |
| AREG | 2.68(2.28-3.46) | 2.44(2.34-2.66) | 0.5267 |
| DAPP1 | 5.74(5.11-5.97) | 5.28(4.68-5.91) | 0.5267 |
| FGF2 | 2.66(1.73-3.69) | 3.11(2.76-3.28) | 0.5604 |
| PSIP1 | 2.99(1.66-4.05) | 1.93(1.32-3.75) | 0.5952 |
| CNTNAP2 | 0.76(0.62-1.05) | 0.76(0.38-1.02) | 0.5952 |
| CLEC4G | 2.06(1.91-2.22) | 1.99(1.82-2.11) | 0.5952 |
| EDAR | 2.45(1.85-2.87) | 2.47(2.04-3.38) | 0.5952 |
| LY75 | 1.08(0.99-1.32) | 1.17(1.11-1.31) | 0.5952 |
| SIT1 | 1.98(1.62-2.50) | 1.85(1.56-2.13) | 0.5952 |
| IL6 | 1.47(1.26-2.47) | 1.60(1.03-3.78) | 0.6308 |
| CDSN | 2.73(2.56-3.05) | 2.61(2.29-3.17) | 0.6308 |
| CD28 | 0.62(0.57-0.77) | 0.57(0.49-0.81) | 0.6308 |
| IRF9 | 2.11(1.19-2.36) | 1.62(1.25-2.25) | 0.6673 |
| PRDX1 | 2.96(2.55-3.11) | 2.97(2.58-3.28) | 0.6673 |
| CKAP4 | 3.90(3.66-4.06) | 3.92(3.73-4.35) | 0.6673 |
| FXYD5 | 0.88(0.84-0.98) | 0.88(0.77-1.34) | 0.6996 |
| KRT19 | 2.71(2.31-3.08) | 2.44(2.34-2.98) | 0.7045 |
| ITM2A | 1.70(1.42-1.95) | 1.64(1.27-1.86) | 0.7045 |
| DFFA | 5.29(4.43-5.76) | 5.04(4.64-5.45) | 0.7045 |
| ITGA11 | 0.91(0.68-1.16) | 0.96(0.57-1.12) | 0.7045 |
| HCLS1 | 4.64(4.27-5.24) | 4.84(4.38-5.18) | 0.7424 |
| MASP1 | 0.39(0.16-0.47) | 0.35(0.23-0.41) | 0.7771 |
| MGMT | 7.28(6.27-7.32) | 7.14(6.80-7.31) | 0.7810 |
| ZBTB16 | 2.73(1.68-3.46) | 2.49(1.99-3.46) | 0.8201 |
| DPP10 | 0.55(0.32-0.77) | 0.56(0.35-0.73) | 0.8201 |
| LILRB4 | 1.79(1.62-2.07) | 1.84(1.74-2.21) | 0.8201 |
| MILR1 | 2.11(1.76-2.29) | 2.06(1.91-2.57) | 0.8201 |
| PTH1R | 3.03(2.84-3.29) | 3.00(2.90-3.38) | 0.8596 |
| CLEC4D | 1.98(1.70-2.16) | 2.00(1.33-2.46) | 0.8596 |
| NCR1 | 1.73(1.46-1.86) | 1.75(1.61-1.90) | 0.8596 |
| IL10 | 2.48(2.25-2.71) | 2.46(2.22-2.81) | 0.8995 |
| SH2D1A | 1.72(0.87-3.10) | 1.78(0.98-3.40) | 0.9396 |
| IRAK4 | 4.21(3.61-4.58) | 4.15(3.90-4.47) | 0.9798 |
| TPSAB1 | 4.74(4.40-5.43) | 4.78(4.29-5.31) | 0.9798 |
| TRIM21 | 2.79(2.36-3.28) | 2.63(2.18-3.23) | 0.9798 |
